# Effect of the Interindividual Variability on Computational Modeling of Transcranial Direct Current Stimulation

**DOI:** 10.1155/2015/963293

**Published:** 2015-07-21

**Authors:** Marta Parazzini, Serena Fiocchi, Ilaria Liorni, Paolo Ravazzani

**Affiliations:** ^1^Consiglio Nazionale delle Ricerche (CNR), Istituto di Elettronica e di Ingegneria dell'Informazione e delle Telecomunicazioni (IEIIT), Piazza Leonardo da Vinci 32, 20133 Milano, Italy; ^2^Dipartimento di Elettronica, Informazione e Bioingegneria (DEIB), Politecnico di Milano, Piazza Leonardo da Vinci 32, 20133 Milano, Italy

## Abstract

Transcranial direct current stimulation (tDCS) is a neuromodulatory technique that delivers low intensity, direct current to cortical areas facilitating or inhibiting spontaneous neuronal activity. This paper investigates how normal variations in anatomy may affect the current flow through the brain. This was done by applying electromagnetic computational methods to human models of different age and gender and by comparing the electric field and current density amplitude distributions within the tissues. Results of this study showed that the general trend of the spatial distributions of the field amplitude shares some gross characteristics among the different human models for the same electrode montages. However, the physical dimension of the subject and his/her morphological and anatomical characteristics somehow influence the detailed field distributions such as the field values.

## 1. Introduction

Transcranial direct current stimulation is a noninvasive brain stimulation technique that utilizes low amplitude direct current to modulate brain excitability, facilitating or inhibiting spontaneous neuronal activity [1]. Its possible applications in clinical neuroscience, as a potential nonpharmacologic, noninvasive, painless, and reversible approach to neurologic disorders, have attracted the interest of many researchers. In the last few years, a lot of clinical studies have been conducted to evaluate the effects of tDCS in the treatment of different diseases, from motor, cognitive, and memory processes to depression and pain syndromes, varying the stimulation parameters and the electrode positions [2, 3]. Recently, this technique has also started to be used in pediatric population [4, 5]. The mechanism of tDCS is believed to arise through a modulation of the baseline cortical excitability, caused by a tonic de- or hyperpolarization of the resting membrane potentials in brain regions experiencing current flow [1–3]. It has been shown that the effects of tDCS depend on the polarity of the electrodes: anodal tDCS has excitatory effect, while cathodal tDCS has inhibitory effects [1–3].

tDCS montages could be classified into different categories according to the position of the reference electrode: intracephalic or extracephalic. Traditionally, the electrodes montage most widely used is the intracephalic, when both of the two electrodes are attached to specific locations on the scalp. To avoid undesirable modulation of the cerebral activity due to the combined effects of the two electrodes on the scalp, it has been proposed to use an extracephalic reference electrode, placed outside the scalp area [1–3], often on the right arm. This position of the reference has been largely used without reporting side effects [6] or safety issues [7, 8].

The increasingly widespread use of this technique and the rising number of clinical applications have boosted the interest in the estimation of the levels of the electric quantities in the brain tissues due to tDCS. This is currently done by electromagnetic computational techniques to guide and optimize tDCS treatment [9]. As a consequence, there has been an increase of publications aiming to quantify the amplitude spatial distribution of the electric field (**E**) and of the current density (**J**) within the human brain tissues during tDCS, by applying various computational methods to different human models, from very simplified head models, like spheres, to MRI-derived head models (for a review, see [9, 10]). Since most of these studies are based on one single head model, it is still unknown whether and how the anatomical differences among individuals may affect the current flow through the tissues and particularly in the brain. This could also have an effect on the outcomes of tDCS treatment.

The aim of this paper is, therefore, to assess the role of the human variability on the amplitude spatial distribution of **E** and **J** within cortical and subcortical brain structures, using realistic human models of different age and gender and considering both the intracephalic and the extracephalic electrode montages. The comparison of the fields estimated in adult with respect to the adolescent will be also discussed.

## 2. Materials and Methods

A commercial simulations platform (SEMCAD X by SPEAG, Schmid & Partner Engineering, AG, Zurich, Switzerland, http://www.speag.com/, [11]) was used to solve Laplace equation (1) to determine the electric potential (*ϕ*) distribution inside a conductive medium due to the stimulation

(1) $$\begin{array}{c} \nabla \cdot \left(\;\sigma \nabla \phi \;\right)=0, \end{array}$$

where *σ* (S/m) is the electrical conductivity of the conductive medium. The distributions of **E** and **J** at every point of the conductive medium were obtained by means of the following relations:

(2) E=−∇ϕ,J=σE.

In any conductive medium with uniform conductivity, **E** and **J** have a linear correlation and their spatial distribution is identical to less than a scale factor (i.e., the conductivity value). Therefore, in the following, we will use the notation “**E** (or **J**)” when we indicate a field characteristics or a quantity which is equal when evaluated from the **E** or **J** distribution in specific tissues, due to their linear correlation.

Three different human models were used in this study, belonging to the “Virtual Population” [12]. These models have been developed from high-resolution magnetic resonance images of healthy volunteers. All models are based on the computer-aided design representation of the organ surfaces, with up to 77 different tissue types represented. A detailed description of the construction of the models is given in [12]. Specifically, we used two adult models of both genders (“Ella” and “Duke”) and one adolescent model (“Billie”), whose characteristics are reported in Table 1.

The dielectric properties of each tissue were assigned using the database [13] built on the data available in literature for low frequency fields [14], with the exception of the skin. This latter was modeled as a weighted average of the electrical conductivities of the skin and of the subcutaneous adipose tissue, which is the tissue just below the skin, following an approach already used in literature [15, 16]. Since the models contain more tissues than what is available in literature [13, 14], we assigned the dielectric properties according to the “recommended tissues' correspondence” of the Virtual Population itself [12].  Table 2 summarizes the conductivities assigned to the tissues [15].

Four clinical electrode montages were modeled: (1)* Montage A*: one electrode was placed on F3 and one on F4, as in [17]; (2)* Montage B*: one electrode was placed on T3 and the reference on the right arm, as in [18]; (3)* Montage C*: two electrodes were placed on C3 and C4, whereas the reference was on the right arm, as in [19]; (4)* Montage D*: one electrode was placed on Fz and the reference on the right tibia, as in [20]. In all the electrode montages described above, F3, F4, T3, C3, C4, and Fz were referred to the 10–20 EEG system.

The electrodes were modeled as a rectangular-pad conductor (*σ* = 5.9 × 10^7^ S/m) of 5 × 7 cm^2^ placed above a rectangular sponge (*σ* = 0.3 S/m) of 7 × 8 cm^2^. The potential difference between the electrodes was adjusted to inject a total current of 1 mA in all the four electrode montages. For each simulation, the human models and the electrodes were inserted in a surrounding bounding box filled with air. The boundaries of the bounding box were treated as insulated; that is, the normal component of the current density was set equal to zero. At the interface between the skin and the air, the current density was set to be parallel to the face.

**Figure 1:**
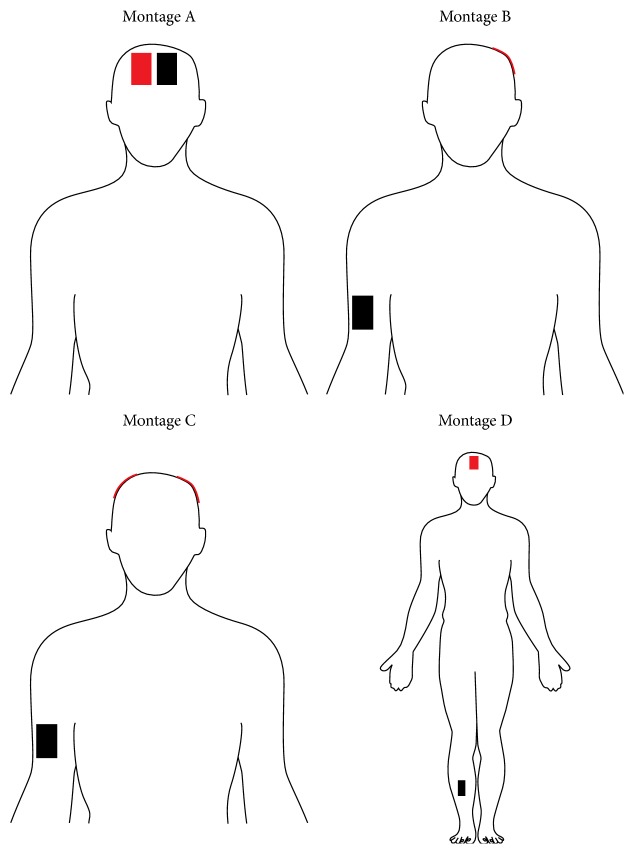
Schematic view of the four electrode montages used here. The red rectangle is the active electrode(s), while the black rectangle is the reference one.

Uniform rectilinear meshes were applied to easily discretize the computational domain with a grid discretization step of 1 mm, with the exception of* Montage D*. In this case, due to the increase of the computational domain, a grid discretization step of 2 mm was used.  Figure 1 shows a schematic view of the four electrode montages.

For all the montages and the human models, the spatial amplitude distributions of **E** (or **J**) were analyzed in different cortical and subcortical brain regions, such as the gray and white matter, the cerebellum, the medulla oblongata, the pons, the midbrain, and the thalamus. Descriptive statistics of the **E** amplitudes (median, 25th and 75th percentiles, minimum, and maximum) were estimated for each brain tissue. For the gray and white matter, we also calculated the percentage of volume where the amplitude of **E** (or **J**) was greater than 70% or 50% of its peak. These percentages of volume will be named in the following sections as* V*70 or* V*50, respectively. The two thresholds of 70% and 50% were arbitrarily chosen, because they correspond to an amplitude reduction of about 3 dB and 6 dB, respectively, with respect to the peak. To assess if there are any significant changes in the percentage of volume* V*70 or* V*50 due to the human anatomical variability, the Kruskal-Wallis one-way analysis of variance by ranks was performed. The* human model* was the only factor with three levels (“Ella”, “Duke,” and “Billie”). If* human model* factor is significant, a Mann-Whitney nonparametric post hoc multiple comparison test, with a Bonferroni adjustment to the criterion of significance, was also performed to assess which pairs of groups (“Ella” versus “Duke,” “Ella” versus “Billie,” or “Billie” versus “Duke”) differ significantly from one another.

The influence of the human variability on the **E** amplitude values was assessed by computing the coefficient of variability (i.e., the ratio between the standard deviation and the mean, expressed in dB) among the models. This was done on both the peak and the median of **E** (*E*_peak_ and *E*_median_) in the cortical and subcortical brain regions, for all the electrode montages.

## 3. Results and Discussion

**Figure 2:**
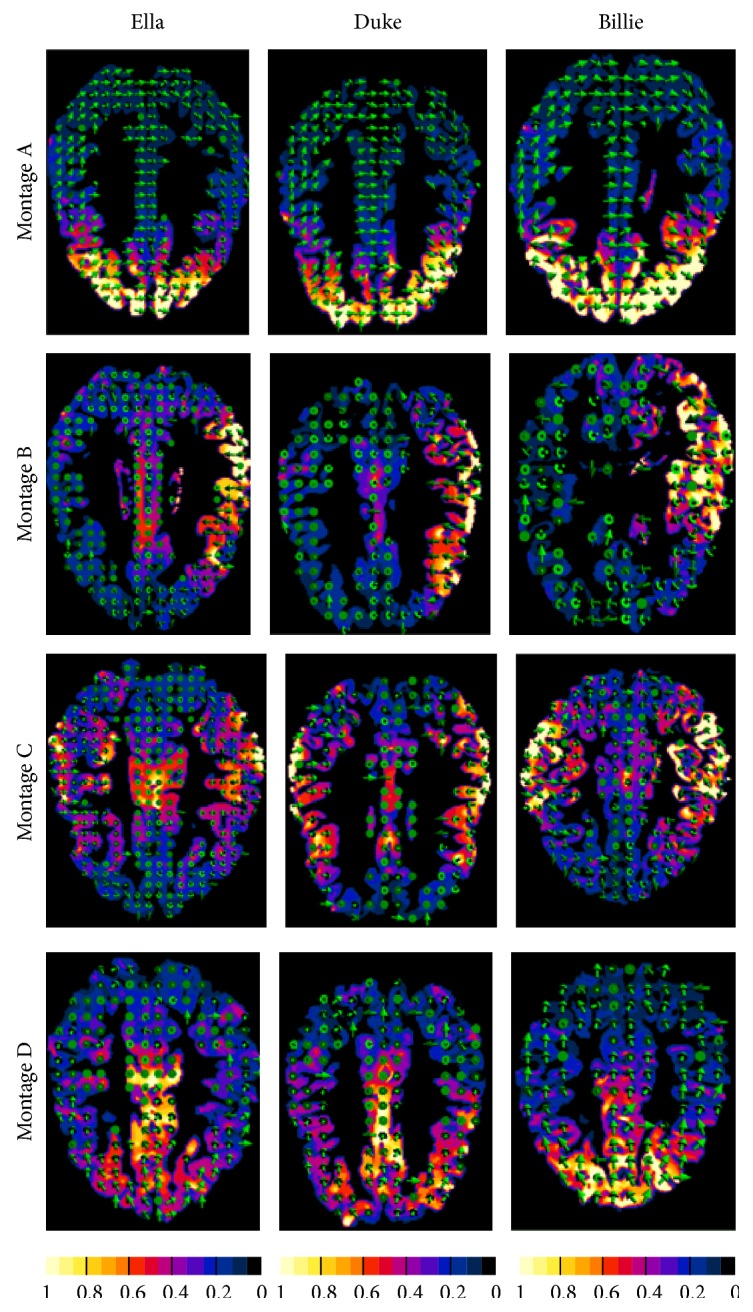
Transversal sections across the gray matter of **E** (or **J**) distribution for all the models for the electrode for Montages A–D. The other tissues and brain regions have been masked on the images. Colour map represents the amplitude of **E**, while the green arrows represent the direction of **E**. The amplitude values are normalized with respect to the peak of the **E** (or **J**) amplitude in the grey matter. Note that those panels can be compared only in terms of spatial distribution but not in terms of amplitude.

Figure 2 shows, as an example, the normalized distribution of **E** (or **J**) on one transversal section across the grey matter for all the models and for the electrode* Montage A* (top row) and* Montage C* (bottom row). Colour map represents the amplitude of **E**, while the green arrows represent the direction of **E**, which, as expected, is mainly directed from the active electrode(s) to the reference one. The maximum value of the color scale is set to the peak of the amplitude distribution in the gray matter for each electrode montage and model. A qualitative comparison of these panels immediately indicates that the general trend of the spatial distributions of the field amplitude shares some gross characteristics among the different human models for the same electrode montages, even if the detailed distributions are complex and depend on the individual head morphology (Figure 2). For all the human models, the symmetric electrode montages (*Montages A* and* C*) tend to have comparable amplitude of **E** (or **J**) in both hemispheres (Figure 2), while the lateralized electrode configuration (*Montage B*) generates higher amplitude of **E** (or **J**) asymmetrically in one hemisphere only. Configuration with at least one of two electrodes on the frontal scalp (*Montages A *and* D*) tends to induce higher amplitude of **E** (or **J**) in the anterior portion of the brain (*Montage A*) and/or in the more central region of the brain (*Montage D*).

To quantify the spread of the amplitude distribution over the gray and white matter, Table 3 reports the percentage of volume where the amplitude of **E** (or **J**) was greater than the 70% (*V*70) or the 50% (*V*50) of its peak, for all the human models and the electrode montages. Data in the table show that varying the electrode montages influences in a similar manner the percentage of volume for any human model. This trend is confirmed by statistical analysis on both these percentages of volume (*V*70 and* V*50), which shows that the* human model* factor was not statistically significant (*P* value > 0.05) for all the electrode montages. The electrode montage that shows the more focalized spatial distribution (i.e., with smaller* V*70 and* V*50) of **E** (or **J**) is* Montage A* for any human model, whereas the electrodes montage characterized by a more widespread spatial distribution of the amplitude on grey and white matter is* Montage C*.

**Figure 3:**
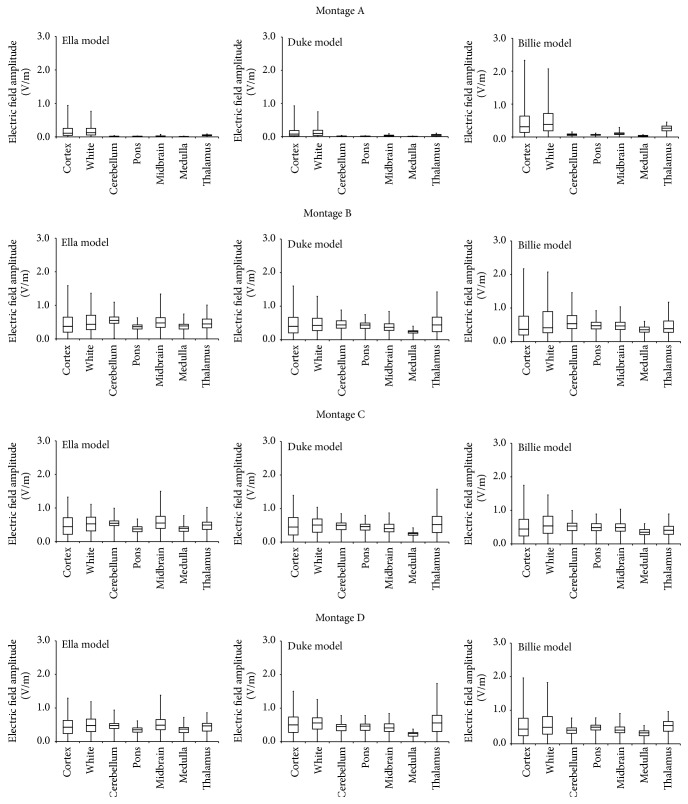
Descriptive statistics of the amplitude distribution of **E** in different brain tissues for each human model, “Ella” (left column), “Duke” (middle column), and “Billie” (right column), across the electrode* Montages A–D* displayed by row. The boxes indicate the interquartile range (25th to 75th percentile) with the median marked by thick horizontal black line. The whiskers delimit the minimum and maximum of the distribution in the specific brain region.

To give a concise but quantitative estimate of the field distributions in cortical and subcortical brain regions, Figure 3 shows the descriptive statistics of the amplitude distributions of **E** evaluated for all the human models and across all the electrode montages. The panels show that “Billie” tends to be characterized by a higher field amplitude with respect to the other adult models, for all the electrode montages. This is particularly true in the cortex and in the white matter and for* Montage A* (Figure 3, 1st row).

**Figure 4:**
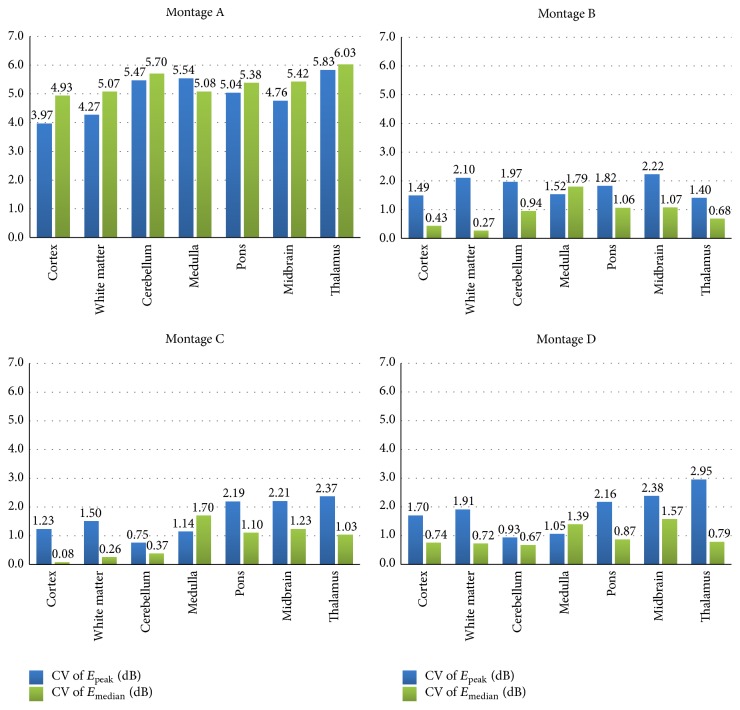
Coefficient of variability (i.e., the ratio between the standard deviation and the mean, expressed in dB) of the peak and of the median of **E** (*E*_peak_ and *E*_median_) due to the variation of the human model for all the electrode montages in different brain tissues.

To quantify the variation due to the human variability on the field amplitudes, Figure 4 reports the coefficient of variability among the models (CV, in dB) of both the peak and the median values of **E** amplitude (*E*_peak_ and *E*_median_), in different brain regions for all the montages. The higher variability (up to almost 6 dB) was found for* Montage A*, while for* Montages B–D* the higher variability was about 3 dB (*Montage D*, thalamus, CV of *E*_peak_). Only for* Montage A*, the CV of *E*_peak_ is lower than the CV of *E*_median_, with the exception of the pons. On the contrary, for all other* Montages B–D*, the variability of the peak is always higher than the one of the median, with the exception of the pons for all the montages.

## 4. Conclusions

The use of tDCS as neuromodulatory technique is rapidly growing [1–3], based on the evidence that delivery of current to specific brain regions can facilitate or inhibit spontaneous neuronal activity. This runs in parallel with the increased use of this technique in pediatric populations for the treatment of various diseases [4, 5].

In this context, it is fundamental to estimate the electric field and/or the current density distributions in specific tissues, by the use of computational techniques on human models. However, the assessment on how the physical dimension of a subject and his/her morphological and anatomical characteristics influence these spatial distributions is not well-known but takes a crucial role in the interpretation of the simulation results.

To this purpose, this study investigates the influence on the **E** (or **J**) field distributions (in terms of both amplitude value and spatial distributions) of the use of realistic models of subjects that differ in gender and age, considering different electrode montages.

Despite some interindividual differences, results of this study show that the broad characteristics of the field distributions due to different electrodes montages are quite similar in all the human models considered here. One example is the spread of the amplitude distribution over the gray and white matter:* V*70 and* V*50 values show no statistically significant difference across the models (see Table 3) and for all the electrodes montages.

However, individual anatomical variability influences the detailed field spatial distributions (Figures 2 and 3), such as the field values. Indeed, both peak and median values of the amplitude distributions of **E** for “Billie” tend to be higher with respect to the two adult models, for the same injected current. This is more evident for the electrode* Montage A* (Figure 3). This is also shown by the higher coefficient of variability found for this electrodes montage on both the peak and the median values of **E** (Figure 4).

The effects on the field distributions found here could be explained by anatomical differences across the models, such as a different cerebrospinal fluid (CSF) distribution, a different skull or fat thickness [9], and a different gray/white matter volume, or by anthropometric variables. For example, since CSF is highly conductive, details of its architecture could profoundly shape current flow through adjacent brain regions [9].

Our results suggest that, to take into account the effect of the human variability on the field distributions in the brain tissue, it could be useful to use some “reference human models” such as “adult” or “children” and “male” or “female,” in the planning of a tDCS treatment. Indeed, during their current practices for brain targeting, clinicians are more interested in a general ranking of which brain regions are characterized by higher field levels instead of the minute details of current flow patterns [21]. Our approach, therefore, could be an alternative to the use of individualized or customized models, to finely tune the tDCS treatment. Although this latter approach is in principle the optimal one [9], building a specific model for each patient can result in an extremely time-consuming and very expensive procedure.

As a final comment, one should note that, in the interpretation of the results of numerical computation of the electric field/current density amplitude distribution, an intrinsic level of uncertainty should be always considered. These uncertainties are difficult to be quantitatively estimated but are qualitatively due to many factors, such as the differences in the used head models (e.g., geometrical models with spheres versus realistic models from MRI images and/or different MRI-derived models), in the head and/or brain tissues considered (in terms of both type and number of tissues), and in the set of dielectric properties of the human tissues applied. Studying this issue in transcranial magnetic stimulation [22], for example, it was found about a 51% of increase of the maximum *E* amplitude when anatomically realistic models are compared to spherical ones. Moreover, as there is no standard protocol for tissue imaging or segmentation, also diversity in the segmented tissue will invariably influence predicted current flow [10]. When an anatomically realistic model is discretized, also the intrinsic level of uncertainty due to numerical artifacts, which are introduced, for example, by the grid resolution, should be taken into account, particularly in the prediction of the peak value [23].

In any case, the numerical computation provides useful information that cannot be obtained experimentally due to the unavailability of methods for high resolution in vivo E-field measurements [24].

## Figures and Tables

**Table 1:** Characteristics of the anatomical models.

Name	Sex	Age (y)	Height (m)	Weight (kg)	BMI (kgm^−2^)	Number of tissues
Ella	Female	26	1.63	59	22.0	76
Duke	Male	34	1.77	72	23.1	77
Billie	Female	11	1.47	35	16.5	75

**Table 2:** Conductivities assigned to the different tissues.

Tissues	Conductivity (S/m)
Adrenal gland, epididymis, hypophysis, pancreas, stomach, stomach lumen, small intestine, small intestine lumen, thymus, thyroid gland, esophagus, and esophagus lumen	0.51113
Internal air, pharynx, and trachea lumen	0
Artery, vein, blood vessel, heart lumen, and penis	0.7
Bladder	0.202783
Bone, mandible, marrow red, patella, skull, teeth, and vertebrae	0.020028
Brain grey matter, hippocampus, hypothalamus, pineal body, and thalamus	0.027512
Brain white matter, anterior commissura, and posterior commissura	0.027656
Breast	0.2617535
Bronchi, bronchi lumen, and ureter-urethra	0.25055
Cartilage, ear cartilage, intervertebral disk, larynx, meniscus, and trachea	0.16113
Cerebellum	0.047512
Cerebrospinal fluid	2
Connective tissue	0.1215635
Cornea	0.4113
Diaphragm and muscle	0.201967
Ear skin and skin	0.012147
Eye lens and ovary	0.3113
Eye sclera	0.501392
Eye vitreous humor	1.5
Fat and subcutaneous adipose tissues (SAT)	0.012207
Gallbladder	0.9
Heart muscle	0.053677
Kidney cortex and kidney medulla	0.0544105
Large intestine, large intestine lumen, and vagina	0.0122052
Liver	0.027714
Lung	0.120847
Medulla oblongata, midbrain, and pons	0.027584
Mucosa	0.0004
Nerve and spinal cord	0.017126
Prostate and testis	0.41113
Spleen	0.0395962
Tendon ligament	0.250922
Tongue	0.26113
Uterus	0.201296

**Table 3:** Percentage of volume of the gray or white matter where the amplitude of **E** (or **J**) is greater than 70% (*V*70) or 50% (*V*50) of its peak for all the electrodes montage and all the human models.

Brain structures	Ella		Duke		Billie	
	*V*70 (%)	*V*50 (%)	*V*70 (%)	*V*50 (%)	*V*70 (%)	*V*50 (%)
Montage A						
Gray	6.6	11.4	6.6	10.7	6.5	11.1
White	8.4	14.4	8.5	13.3	8.5	14.6
Montage B						
Gray	8.6	17.5	8.3	18.1	7.9	15.3
White	12.6	26.5	10.1	24.5	11.6	20.6
Montage C						
Gray	9.9	29.2	10.2	28.4	7.0	17.6
White	19.5	46.7	20.3	48.4	13.4	32.0
Montage D						
Gray	8.6	23.4	9.5	23.8	8.4	17.0
White	11.8	33.3	12.2	37.3	11.5	20.8
